# Expanding the *Miscanthus* market in the UK: Growers in profile and experience, benefits and drawbacks of the bioenergy crop

**DOI:** 10.1111/gcbb.12997

**Published:** 2022-09-14

**Authors:** Rebecca von Hellfeld, Astley Hastings, Jason Kam, Rebecca Rowe, John Clifton‐Brown, Iain Donnison, Anita Shepherd

**Affiliations:** ^1^ School of Biological Sciences University of Aberdeen Aberdeen UK; ^2^ Terravesta Lincoln UK; ^3^ UK Centre for Ecology & Hydrology Lancaster Environmental Centre Lancaster UK; ^4^ Institute of Biological, Environmental & Rural Sciences (IBERS) Aberystwyth University Aberystwyth UK; ^5^ Department of Agronomy and Plant Breeding I University of Giessen Gießen Germany

**Keywords:** BECCS, bioenergy policy, decarbonisation, grower survey, *Miscanthus*, net zero strategy, sustainable agriculture

## Abstract

To achieve net zero greenhouse gas emission by 2050 as set out by the 2019 amendment to the 2008 UK Climate Change Act, a major shift towards renewable energy is needed. This includes the development of new methods along with improving and upscaling existing technologies. One example of new methods in bioenergy is developing new *Miscanthus* cultivars for electricity generation via thermal power station furnaces. *Miscanthus* is still relatively new compared with other agriculture practices, so market assessments and improvements are needed to reduce the barriers to entry for prospective growers. This publication provides a profile of UK *Miscanthus* growers and their businesses, their experiences of benefits and drawbacks of the crop, and what they see as potential barriers to entry for prospective farmers. A survey of current *Miscanthus* growers in England and Wales was conducted and indicated that most farmers were content with the crop and that its environmental and economic benefits were noted. However, it was evident that with a geographically limited UK market, growers wanted to see a better distribution of biomass processing stations to reduce the ongoing costs of transport. With growing demand for renewables, including bio‐energy sources, it was determined important to provide information and support for stable farming operations and to incentivise the adoption of *Miscanthus*. Such incentives include ongoing development of new cultivars, focussing on traits such as production potential and stressor resilience, and growers indicated preference for an annual planting grant. These developments are predicted to further improve the crop's profit margin, making it a more cost‐effective crop for farmers. Sensitively managed *Miscanthus* also has the potential to contribute to carbon sequestration, soil health, and aspects of farmland biodiversity. Incentivising such management in government land–based environmental schemes would offer additional income streams and help to promote environmental positive crop planting.

## Graphical abstract

This paper outlines the profile and experience of Miscanthus growers and development needs to upscale the UKs Miscanthus crop area in accordance with the industry requirements, to achieve the country’s net zero target by 2050. We found that current growers are satisfied with the crop, but long‐term stability is required to incentivise prospective growers. Moreover, the network of processing facilities needs to improve to reduce transport costs, and recognition of the crop’s role in reaching the UK CCC targets is needed, as well as information on the other ecological benefits it provides.

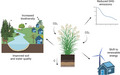

## 1 INTRODUCTION

Ideal bioenergy crops are perennial, such as the Elephant grass (genus *Miscanthus*), use resources effectively (e.g., being water‐ and nutrient use efficient), require low to no addition of fertilisers, contribute to soil carbon storage, and are non‐invasive (Heaton et al., 2004). *Miscanthus* has a life span of up to 20 years and although it originated in Eastern Asia, some species have developed cold tolerance, allowing it to thrive in temperate climates (Clifton‐Brown et al., 2015; Finch et al., 2009; Karp & Shield, 2008). One such species is *Miscanthus* × *giganteus* (referred to as *M* × g; herein referred to as *Miscanthus*), a hybrid between *Mischanthus sinensis* and *Mischanthus sacchariflorus* (Carroll & Somerville, 2009). This hybrid is a highly productive, rhizomatous perennial grass that is non‐invasive, due to being sterile (Anderson et al., 2011). Once the crop is established, it can grow up to 4 m tall in one growing season. The harvested dry mass in the United States can be up to 40 tonnes per hectare per year (t ha^−1^ year^−1^) and yields in temperate Europe average 19 t ha^−1^ year^−1^, and 12 t ha^−1^ year^−1^ in the United Kingdom (Harvey, 2007; Lewandowski et al., 2003; Shepherd et al., 2020). Compared with other perennial bioenergy crops such as short rotation coppice (SRC) willow, *Miscanthus* yields a greater dry matter content, albeit providing a lower energy content than SRC willow (DEFRA, 2019a; Hastings et al., 2014). *Miscanthus* also sequesters carbon and can grow on marginal lands and unused fields (Carroll & Somerville, 2009; McCalmont et al., 2015; Milner et al., 2016). Other benefits include that *Miscanthus* does not require fertiliser use after establishment, and no field management other than the annual harvest (Shepherd et al., 2020).

*Miscanthus* has been cultivated for combustion in Europe since 1935 and has been part of the agricultural landscape of the United Kingdom (UK) since the 1990s (Clifton‐Brown et al., 2015). In the United Kingdom, bioenergy is generated from *Miscanthus*, with the crop usually dried, chopped, and packed into bales for firing in power station furnaces although it can also be pressed into pellets or briquettes for use in domestic or larger boilers and burners (Terravesta, 2022). While having some use outside of the bioenergy market, such as livestock bedding, phyto‐remediation, construction, insulation, reinforcing fibres and domestic fuel, it has a greater efficiency for energy generation in comparison with first‐generation crops such as sugar beet, corn or rapeseed and a higher greenhouse gas (GHG) mitigation potential, due to its' lower input requirements (Don et al., 2012; Karp & Shield, 2008; McCalmont et al., 2015). Even with these evident benefits, *Miscanthus* accounts for less than 1% of the UK's bioenergy crop use, with the rest being accounted for by wheat and maize mostly, as well as sugar beet and SRC (DEFRA, 2019a).

**FIGURE 1:**
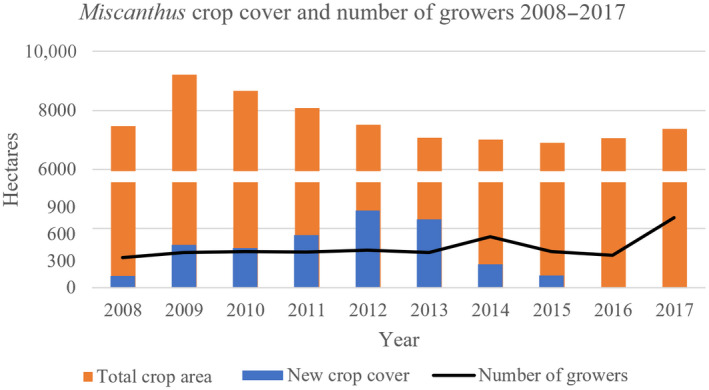
Number of new *Miscanthus* growers (black line), annual total area covered by *Miscanthus* crop each year (orange bar), and portion of that constituting newly planted crop (blue bar) in England, Wales, and Ireland between 2008 and 2017. Data obtained from DEFRA (2019a).

It has been determined that, when excluding all unsuitable and restricted lands, the United Kingdom has a maximum of 8.5 million ha of land that could potentially be available to grow bioenergy crops (Lovett et al., 2014). As of 2021, however, the total land coverage for *Miscanthus* in the United Kingdom makes up less than 0.1% of that (~10,000 ha). Incentives to increase dedicated energy crops (DECs) such as SRC and *Miscanthus* in England, Wales and Ireland have included the two Energy Crop Schemes (ECS) which ran from 2000 to 2006/2007 (ECS1) and 2008–2015 (ECS2), supporting farmers with, for example, establishment costs (Glithero et al., 2013), but not with ongoing costs like harvesting and transport (Thornley, 2006). The second ECS (ECS2) successfully incentivised growers to switch to *Miscanthus*, leading to an increase from just more than 300 growers in 2008 to more than 400 in 2015. Even after the end of the ECS2, however, the number of *Miscanthus* growers in the United Kingdom continued to rise, reaching almost 800 in 2017 (Figure 1). ECS2 had a substantial effect on enabling farmers to grow *Miscanthus*, as well as improving the machinery and investing in research to improve the output (Terravesta, 2021). The efforts of the UK government and the UKRI to fund research have facilitated and improved breeding, agronomy, and harvesting with projects like GIANT‐LINK (http://www.miscanthusbreeding.org/results.html), which make the crop more acceptable for farmers. It was found that the most common reason for growers to invest into *Miscanthus* were not based on the financial return, but instead related to the low requirement for field operations, low maintenance cost, and regeneration capacity. This provides a practical solution for fields that are difficult to access with social acceptance near public fields, as well as allowing to use the fields for gamebird cover, providing a secondary income. Thus, *Miscanthus* provides a solution to practical problems while improving the environment (Shepherd et al., 2020).

With the current policy to reduce GHG emissions and to invest in carbon capture and sequestration methods (e.g., through the Carbon Capture and Storage Infrastructure Fund (CIF); BEIS, 2021b), the focus is shifting to increasing *Miscanthus* as a crop for bioenergy (BEIS, 2021a; CCC, 2019; Clifton‐Brown et al., 2017) and also now for GHG removal. The UK bioenergy power stations which use *Miscanthus* today were initially designed to burn excess cereal straw, but there is a preference for *Miscanthus* fuel, due to better combustion properties. However, the annual *Miscanthus* production of ~100,000 t is insufficient to meet the ~500,000 t annual fuel requirement of such power stations (Harvey, 2007). Based on the Climate Change Act (UK Government, 2008, 2019), the United Kingdom is required to achieve a net 100% reduction in GHG emissions by 2050 compared with 1990 levels. Now that the United Kingdom has left the European Union (EU), the United Kingdom is no longer tied to Common Agriculture Policy (CAP) regulations and is currently formulating its own land use and farm incentive plan called the Environmental Land Management Scheme (ELMS) in England, which is likely to include bioenergy crops in the climate mitigation and adaptation elements of this policy (Cross et al., 2021). Schemes like ELMS are also in progress in the devolved nations. *Miscanthus* reduces fertiliser need due to its high nitrogen (N) recycling efficiency and hence has a reduced nitrous oxide (N_2_O) emission. *Miscanthus* is thus considered to be an environmentally sustainable crop for inclusion in ELMS (Don et al., 2012).

### 1.1 Aims and objectives

Conventionally, *Miscanthus* has been propagated on from rhizomes in rhizome‐nursery fields via in vitro propagation—a relatively slow and expensive process, which meant a delay to provide growers with plants and a limited ability to increase planted area (Clifton‐Brown et al., 2017). Seed‐propagated hybrids of *Miscanthus* facilitate a more rapid upscaling of the crop, which will be essential for meeting demanding decarbonisation targets. With new hybrids of *Miscanthus* ready to be introduced to the market, and the need for meeting net zero targets, an expansion of *Miscanthus* growers is needed. This research is aimed at understanding the motivation of farmers to grow this new crop as well as to understand the barriers to adaptation, informing strategies to attract new growers.

This research examines the current situation of *Miscanthus* growers in the United Kingdom, as well as establishing the needs that must be met to ensure the successful future expansion of *Miscanthus* growers. The work aims to develop a profile of the typical *Miscanthus* grower and their farm through targeted questions in an anonymous survey. Questions about the perceived drawbacks and benefits of the crop provide insight into the aspects of the cooperation between growers and the industry that require strengthening. The results of this survey are also assessed considering previously conducted works to determine potential changes in perception over time.

## 2 MATERIALS AND METHODS

### 2.1 Grower questionnaire

The grower's questionnaire was sent out to all Terravesta‐contracted *Miscanthus* growers in England and Wales in August of 2021. The questionnaire contained 37 questions (Material S1) with initial questions about the farm, followed by questions about the grower's demographic status and experience in the sector (age, education, involvement in green schemes, etc.), followed by questions on their experience of the benefits and drawbacks of growing *Miscanthus*. The survey included multiple‐choice and open‐ended questions, with multiple‐choice responses based on the 5‐point Likert scale: ‘strongly disagree’ (score 1), ‘disagree’ (2), ‘neutral’ (3), ‘agree’ (4), and ‘strongly agree’ (5). The statements were based on the outcome of a previous grower's survey by Shepherd et al. (2020) and discussion with stakeholders and other researchers associated with the UKRI funded Supergen Bioenergy Hub.

The questionnaire was created in Snap 11 (Version 11, build 11.22), which is managed online by the Snap WebHost. The questionnaire passed the research ethics review of the University of Aberdeen and a link to the questionnaire was distributed by Terravesta in a newsletter sent to their *Miscanthus* growers. The questionnaire was open until the October 31, 2021. Although the participation remained anonymous, interested parties were offered the opportunity to access a separate prize draw link at the end of the questionnaire as an incentive to respond to the survey.

Each grower was asked to answer the questionnaire, only two questions (questions 1 and 21) were mandatory, and all others were voluntary (Material S2). The analysed results were given as a percentage of responses, also accounting for missing responses. Where a limited number of response options were provided, a following question allowed for free input to provide other responses that were not included in the list. Descriptive statistics were calculated to summarise the survey data. Responses to open‐ended questions were captured in a table. Data visualisation used Excel (Version 2202, build 16.0.14931.20118).

## 3 RESULTS

### 3.1 About the farm

**FIGURE 2:**
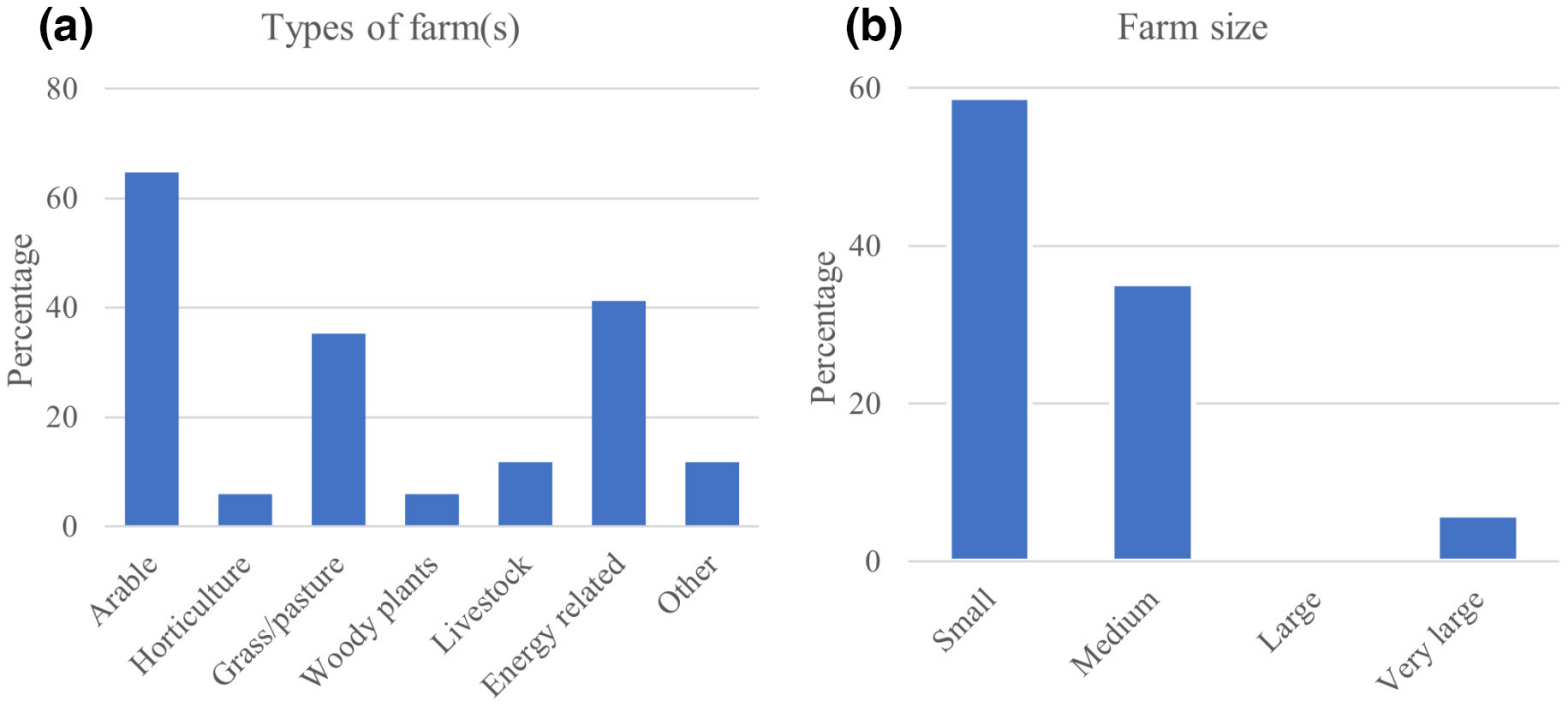
Type (a) and size (b) of the farms owned by the respondents, presented as percentage of total answers (*n* = 17). For the farm type, multiple responses could be given. For the farm size, small is defined as having a labour requirement of less than two full‐time workers, medium as requiring at least two but less than three full‐time workers, large as requiring at least three but less than five full‐time workers, and very large as requiring five or more full‐time workers.

A total of 17 current Terravesta *Miscanthus* growers responded to the survey, and the raw data of all responses can be found in Material S2. Growers were asked their type of farm, and allowed to respond with more than one type. Most farms were arable (65%), energy related (41%), and/or pasture based (35%; Figure 2a). Only six farms were solely dedicated to arable farming, and one to each pasture, horticulture, and energy‐related farming. The remaining eight farms were a mix of different types of farming operations. Almost 60% of surveyed farms were small businesses (labour for less than two full‐time workers as defined by the Farm Business Survey for England, Wales, and Northern Ireland; DEFRA, 2022), and less than 40% were medium sized (labour for 2–3 full‐time workers; Figure 2b).

**FIGURE 3:**
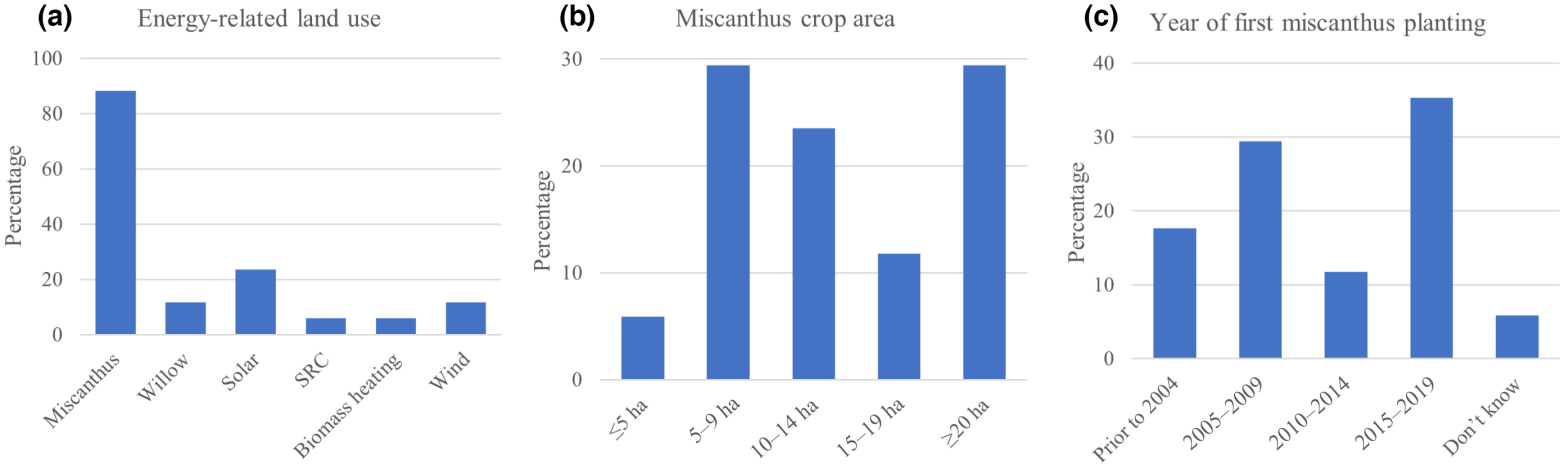
Type of energy‐related farm use (a), *Miscanthus* crop area (b), and year of first *Miscanthus* planting (c), presented as percentage of total responses (*n* = 17).

A total of 88% of energy‐related activities were *Miscanthus* crop, followed by 24% using solar power, and 12% each for willow and wind power. 6% also responded with biomass heating and SRC (other tree species than willow; Figure 3a). Excluding very small areas of under 5 ha *Miscanthus* crop area produced a “U” shaped distribution with most growers either having small 5–9 ha or large areas >20 ha (29% in both cases) farm (Figure 3b). For most farms presented here, *Miscanthus* made up between 5% and 40% of area cover, with only one farmer having less than 2% *Miscanthus* cover, and one solely farming *Miscanthus* (data not shown, for details see Material S2). Most respondents started growing a *Miscanthus* crop during the period 2015–2019 (35%), followed by 29% during the period 2005–2009, followed by 18% before 2004, and 12% during the period 2010–2014 (Figure 3c).

**FIGURE 4:**
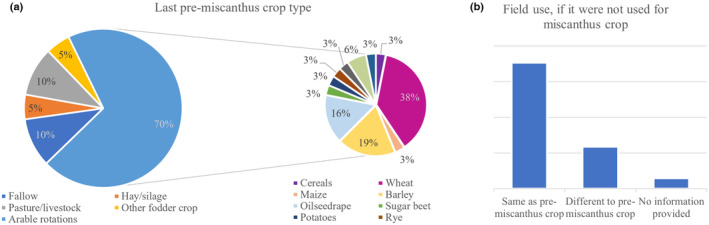
Crop grown in fields now used for *Miscanthus* prior to first planting it (a) and use of the field, if *Miscanthus* had not been planted (b) presented as percentage of total answers (*n* = 17).

About 70% of participants stated that before planting *Miscanthus* the fields were used for arable rotations (Figure 4a). Of these, almost 40% grew wheat during their last rotation prior to planting *Miscanthus*, whereas barley and oilseed rape were the second and third most previously planted crop (16% and 19%, respectively). When asked what would currently be growing on the field, if they had not planted *Miscanthus*, more than 70% of respondents stated they would have used the field(s) the same way they did in the year before planting *Miscanthus*. Only one participant did not provide any information to this question (Figure 4b).

**FIGURE 5:**
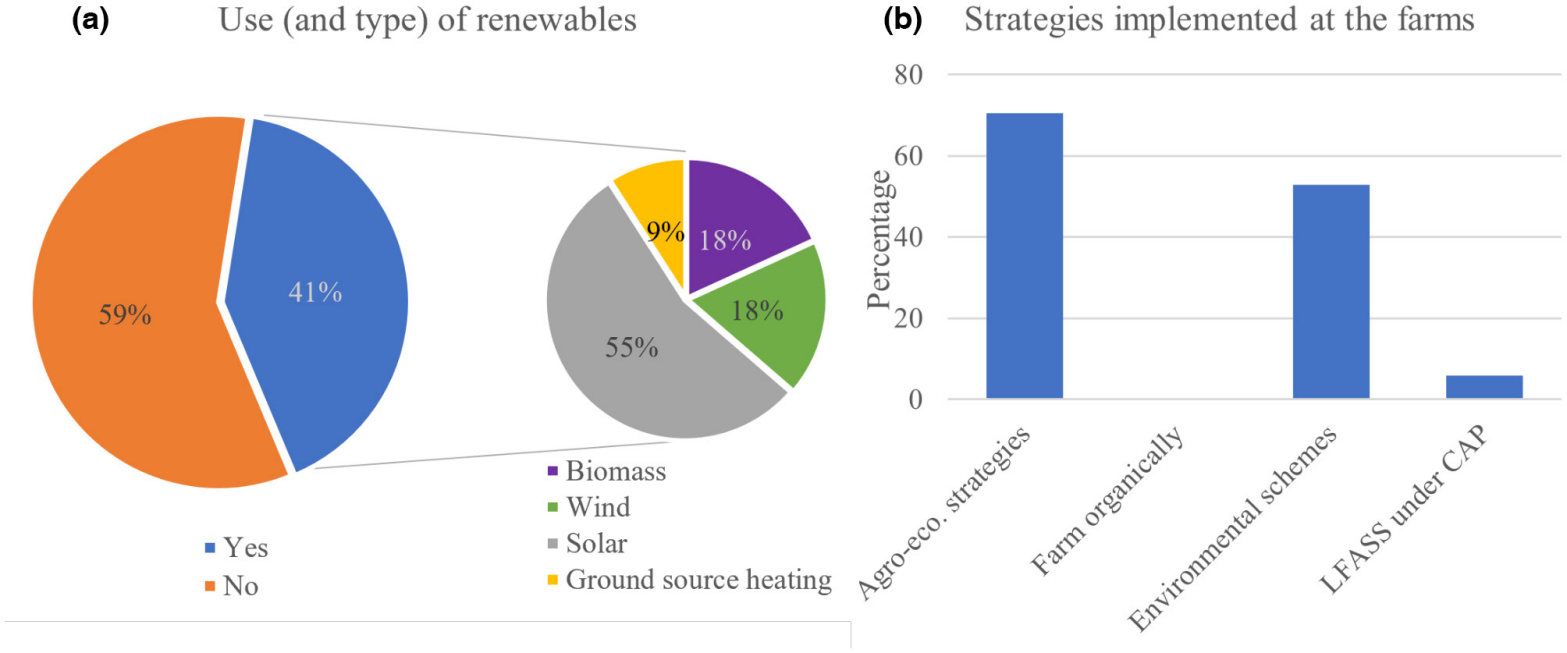
Type of environmental strategies implemented at the farm (a) and use of renewable energy (b) presented as percentage of total responses (*n* = 17).

Although none of the farms were organic, more than 70% of participants implemented agri‐ecological strategies at their farm, and more than 50% were part of environmental schemes (Figure 5a). In addition, more than 40% of the respondents used renewable energy for the farm, of which 55% was solar energy (Figure 5b). Other forms of renewable energy used were wind power (18%), biomass (18%), and ground source heating (9%).

### 3.2 About the grower

**FIGURE 6:**
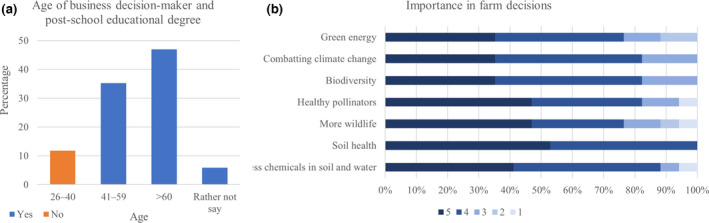
Age of the business decision‐maker (a; percentage of individuals per age bracket without a degree shown in orange). The importance of sustainable agriculture options to the business decision‐making process (b; with responses for each option given from 5 [very important] to 1 [not important at all]).

In the survey participants, the typical business decision‐maker was >60 years old, followed by 41–59 years old, and only 10% were 26–40 years old. Additionally, results showed that only two of the participants had no post‐school educational certificate or degree, both of which were <40 years old (Figure 6a).

When asked about the importance of different sustainable agriculture‐related aspects to the decision‐making process of the farm (Figure 6b), it was found that most of these issues were considered as relevant or very relevant (response levels 4–5). “More wildlife in the fields” and “less chemicals in water and soil” were considered to being the most important factors for decision‐making, although 30%–50% of the respondents said that all listed options were very important (response level 5) or important (level 4).

### 3.3 Benefits and drawbacks of *Miscanthus*

**FIGURE 7:**
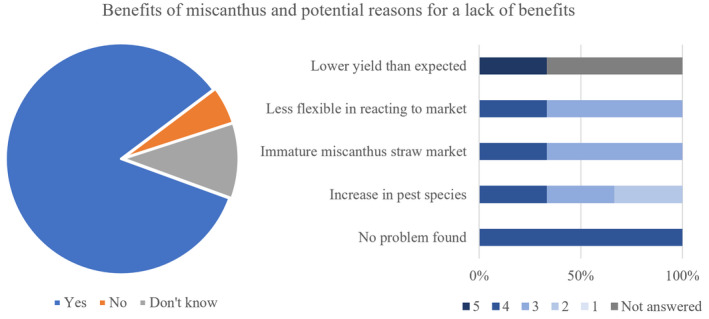
Responses to whether *Miscanthus* has a benefit on the farm and possible reasons for a lack of benefits. For the importance of the drawbacks, responses for each option given from 5 (very important) to 1 (not important at all).

When questioned on the drawbacks of *Miscanthus*, only one participant found *Miscanthus* not to benefit the farm, whereas two respondents were unsure and the remaining 14 farmers found the crop to benefit the farm. The three respondents who answered “no” or “I don't know” were then asked for potential reasons, with the most relevant option being that there was no problem with the crop, but no benefit either. Only one of those who qualified for this question felt that the lower than expected yield was a highly relevant drawback (response level 5), whereas the remaining two growers did not respond. The remaining potential drawbacks were perceived as a relevant (4) drawback by one third of respondents, whereas the remaining two thirds were neutral (3) or considered it less relevant (2) (Figure 7).

**FIGURE 8:**
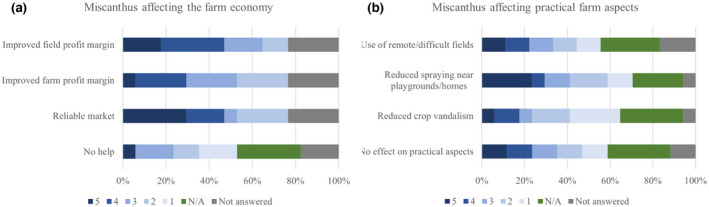
Responses to how *Miscanthus* affects the farm economy (a) and practical aspects (b). For the importance of the effects, responses for each option given from 5 (very important) to 1 (not important at all).

When asked about the economic benefits of *Miscanthus*, the most common response (29%) was that the reliability of the market, and the improved profit margin of the fields growing *Miscanthus* were the most relevant benefits (Figure 8a). Overall, most responses for the provided options were between response levels 5 and 2, with only the option of *Miscanthus* having no overall effect on the farm economy being not applicable at all (1). Other reasons provided for improving farm economy were reduced workload, reduced need for hired labour, the use of small fields, the spreading of risk because of a guaranteed market, the low maintenance requirements of the crop, and that the crop was good on wet heavy land (Table 1).

**TABLE 1:** Quotes and paraphrasing of answers given to questions regarding the farm economy and practical aspects (questions 24–27), barriers to extending *Miscanthus* crops currently (questions 22–24) and the main characteristics that may improve *Miscanthus* production in the United Kingdom in the future (question 36)

Benefits to the farm economy	Benefits to practical aspects	Barriers to extending *Miscanthus* area	Improving *Miscanthus* production in the United Kingdom
Freed up workload	It requires less input than other crop	Landlord/shoot	Recognition of the crop as being carbon negative (capturing/storing carbon) and monetising the capture/sequestration
Use of small fields	It stopped annual soil erosion of sandy soil	Changing contracts and payment delays	More outlets for the crop are needed
Reduced need for hired labour			The ignorance to the crops' advantages
			It is not mentioned by DEFRA/RPA
Crop is good on wet heavy soil	It reduced run‐off after heavy rains	Cost of road haulage	Most farmers have no first−/second‐hand experience with the crop and are wary of switching
Spreads the risk due to a guaranteed market	It is entirely contractor/rent‐based, which is beneficial for inexperienced growers	With a limited market, some regions experience higher transport costs	Government incentives or subsidies are needed
			More local markets
			Increased bale prices and a less complicated price formulation
It is a low maintenance crop once established	It reduced the need for chemicals and fertilisers	Research for alternative uses and more distributed processing would be required	Research on long‐term soil health benefits
			Agronomic steps to extend maximum yield over increase lifespans to reduce replanting needs
			A larger selection of high‐yield variants for different soil conditions

The reduced need for spraying near playgrounds and homes was the most common ‘very applicable (5)’ response when asked about the benefits of *Miscanthus* in practical aspects of the farm (Figure 8b). A more homogenous distribution of votes for all four response options between 5 and 1 was observed for this question. Additional reasons for the practical benefits of *Miscanthus* were the reduced input requirements, the contract‐based collaboration being beneficial for inexperienced growers, reduced annual soil erosion of blow‐away sand, reduced need to use chemicals and fertilisers, and the reduction of run‐off of heavy rain and soil (Table 1).

**FIGURE 9:**
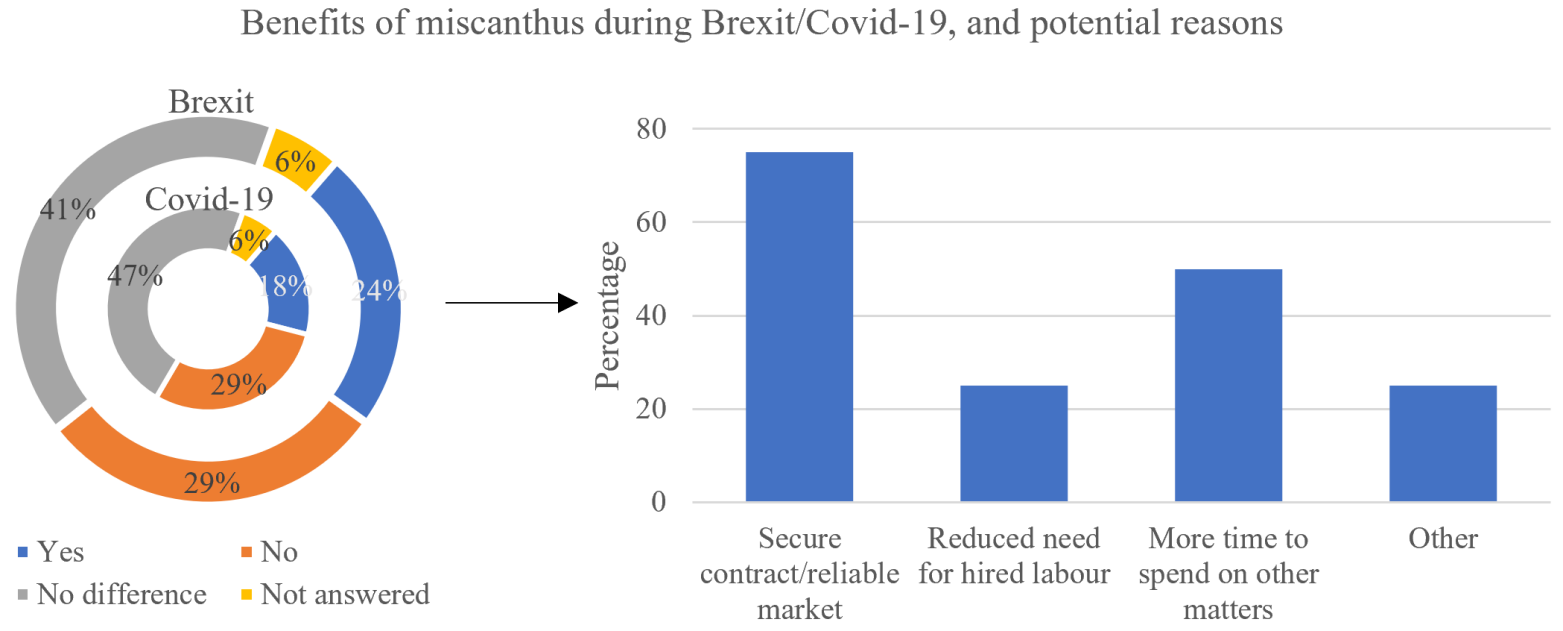
Potential benefits of *Miscanthus* during Brexit/Covid‐19 and the potential reasons for these perceived benefits.

Most farmers did not notice a difference or a positive effect of *Miscanthus* during Covid‐19 (47% no difference and 29% no, respectively) or Brexit (41% no difference and 29% no, respectively). One participant did not respond (Figure 9). Of those who noted a positive impact of growing *Miscanthus* during Brexit (*n* = 2) or Covid‐19 (*n* = 4), 75% stated that the secure contract and reliable market were the reason for the noted benefits, whereas 50% responded that the freed up time was beneficial, and 25% also stated that the reduced need for labour was helpful.

When asked about practical difficulties with growing *Miscanthus*, 35% of participants felt that they had not received enough information about *Miscanthus* crop growing prior to the first harvest, and only three respondents said they removed *Miscanthus* crop from their field and found it difficult (Material S2).

**FIGURE 10:**
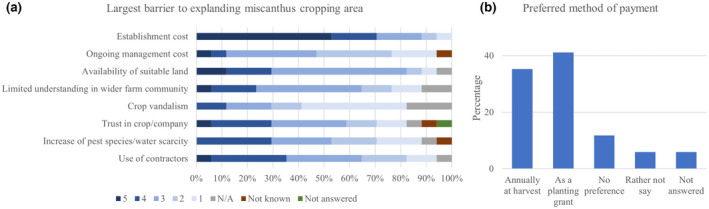
The barriers to entry for expanding the *Miscanthus* crop area (a) and the preferred payment method (b). For the importance of different barriers, responses for each option given from 5 (very important) to 1 (not important at all).

When asked about the barriers to expanding the current *Miscanthus* cropping area, most respondents felt that the cost of establishing the crop was the greatest barrier (response level 5: 53%, 4: 18%, and 3: 18%). This was followed by the lack of suitable land available for the crop (5: 12%, 4: 18%, and 3: 53%), the use of contractors (5: 6%, 4: 29%, and 3: 39%), and the potential lack of trust in the crop or company (5: 6%, 4: 24%, and 3: 29%). With 41% of participants stating it was not important at all, crop vandalism was the option that least limited the crop expansion (Figure 10a). Other barriers to entry included the cost of road haulage, the limited market, and changing contracts (Table 1).

When asked in what form any financial incentive should take, the grower's preference for payment method was an annual planting grant (41%), the second most popular was an annual payment at harvesting (35%). 12% of growers had no preference for payment method and one participant in each case preferred not to respond or did not answer (Figure 10b). Of all participants, 71% further stated that they would consider expanding their *Miscanthus* crop in the future, whereas 18% were not sure, and only 12% answered with ‘no’ (Material S2).

The main characteristics that growers believe would improve the production of *Miscanthus* in the United Kingdom are listed in Table 1. The need for government incentives or subsidies was stated, as well as the need for the government to recognise, and monetarise, the carbon capturing and sequestration benefits of *Miscanthus*. The responses also indicated that there was a general lack of information regarding the short‐ and long‐term benefits of *Miscanthus*, which might be limiting potential growers from entering the market, and that the market overall was not ready for large‐scale *Miscanthus* operations.

## 4 DISCUSSION

With the readiness to market of a new seed‐propagated variety of *Miscanthus*, promising a greater upscaling potential and resilience, the industry capacity to expand through, for example, an increased availability of rhizomes, plant plugs and seeds, needs an increased number of growers willing to plant the crop. This research has highlighted the motivation and barriers to farmers to adopt *Miscanthus* as a bioenergy crop. With increasing demand for renewable energy, including bioenergy crops such as *Miscanthus* (BEIS, 2022), the need for farmers to enter the market must now be considered, to successfully upscale the UK's *Miscanthus* crop and bioenergy sector. In this work, we sought to support this endeavour by profiling UK *Miscanthus* growers and identifying barriers to entering the market, to help the government and industry better understand the needs of prospective farmers and to determine who would be most likely to switch to *Miscanthus* in the future.

### 4.1 Survey responses in context

The demographic profile of *Miscanthus* growers surveyed in this research show that most business decision‐makers are >60 years old and have a post‐school degree. The average farm size for the surveyed farms is 223 ha (Material S2) and require less than two full‐time workers (Figure 2). To determine how representative the survey respondents are for the average UK farm, the responses were put in context of the 2019 ‘future farming and environment evidence compendium’ (herein future farming statistics) published by DEFRA (2019b). The age profile and labour requirement of the survey respondents reflected distributions seen in national statistics presented in the future farm statistics, with the national average UK farmer being aged 60+ and a full‐time work force requirement of two for all non‐horticulture operations (DEFRA, 2019b). In addition, none of the farms presented here identified as organic (Material S2), which makes them representative of most UK farms (97%). However, the average farm size of respondents is above the national average of 87 ha (DEFRA, 2019b), and 71% of respondents stated a farm area exceeding the national average.

Of the 17.4 million hectares of land used for the agricultural industry in the United Kingdom, the predominant farmed area is grassland, only around 30% of this is accounted for by croppable area (DEFRA, 2019b). If the 17 respondents of this survey represent an average cross‐section of the current *Miscanthus* growers (Figure 2a), their predominant previous land use being arable, further supported by the findings of a previous survey (Shepherd et al., 2020), this would suggest that livestock farmers are not as likely to convert to *Miscanthus*.

Most farms presented here either have between 5 and 9 ha of *Miscanthus* crop, or more than 20 ha (Figure 3b). Although the total area of *Miscanthus* in England between 2008 and 2017 is between 6905 and 9213 ha, the average areal coverage per grower falls between 9.36 and 23.41 ha (DEFRA, 2019a). Thus, around 30% of farmers presented here, have an above average crop area for *Miscanthus*, compared with the national average. Most respondents stated that their *Miscanthus* fields had a previous arable land use with crop rotations. The future farming statistics outline that more than 40% of the current used agricultural area is accounted for by arable crops, with permanent grasslands being the only larger sector (DEFRA, 2019b).

### 4.2 Environmental drivers for the adoption of *Miscanthus*

Respondents were asked whether environmental considerations impacted their decision grow *Miscanthus*. Although this was not scaled against alternative factors, such as financial reward, all growers noted that positive environmental impacts were factors in the decision‐making. Of the environmental impacts considered soil health was the most important contributing factor in this decision‐making. With 33% of UK soils being degraded, and more than 1 mil. ha being at risk of erosion (DEFRA, 2019b), the fact that soil health was among the most important contributors to farming decisions being made (Figure 6b) is representative of farmer awareness of this condition. This might be explained by the fact that Terravesta actively seeks out the least productive field(s) of a farm, highlighting the regenerative benefits of *Miscanthus* (pers. Com.).

In 2020, the United Kingdom developed a 10‐point plan for a green industrial revolution, which includes addressing usage, carbon capture and storage (UCCS) processes (HM Government, 2020). On a small scale, a payment programme for carbon captured in soils has already been developed by Soil Capital, offering a minimum of £23/t of carbon dioxide equivalent for improvements that reduce GHG emissions as well as increasing carbon storage in soils (Abram, 2021). The latter would include *Miscanthus*, known for its soil carbon storage ability (see e.g., McCalmont et al., 2015). Additionally, the nitrogen recycling ability of the crop means that less GHGs are being created through nitrification. This further supports the UK's aim to reduce the nitrate pollution (House of Commons, 2018).

### 4.3 Use of other renewables

Additionally, around 21% of UK farms carry out solar energy operations, and 10% use other sources of renewable energy (DEFRA, 2019b). Overall, 41% of the farmers presented here used some form of renewable energy, with solar energy accounting for 55% of those that do (Figure 5b). Thus, it could be theorised that many growers who are investing into bioenergy crops such as *Miscanthus* are more inclined to also get involved in other green energy or environmental schemes for alternative energy sources for farm use. This is often made more accessible by companies such as Solarsense, offering asset finance support and competitive power purchase agreements (PPAs) for farms to make use of solar energy (Solarsense, 2022). PPAs lower the cumulative energy costs, providing an additional incentive for using renewable energy sources (World Bank Group, 2021). Additionally, offers such as the West of England Green Business Grant for small and medium businesses allow for the coverage of 40% of eligible costs for the installation of commercial solar panels (WECA, 2020). These show direct and long‐term incentives for farmers that are also needed for bioenergy crops such as *Miscanthus*.

### 4.4 Barriers identified

From the survey some of the barriers to taking up *Miscanthus* crop were established (Figure 10a). The survey responses mentioned lack of trust in the crop, which has previously been described as an uncertainty around the financial returns on the crop in comparison with arable crop returns (Adams et al., 2011; Sherrington et al., 2008; Thornley, 2006). Although none of the participants of the survey presented here noted that the limited market itself was an issue, the resulting road haulage costs were noted as a barrier (Table 1). ECS1, aimed at increasing the amount of energy crop grown in the United Kingdom, addressed establishment cost only by providing a fixed rate payment per hectare, covering 40%–50% of the costs for establishing energy crops such as *Miscanthus* (DEFRA, 2003) but ignored the need for an established market. The second ECS was launched in 2008, also covering 50% of establishment costs. However, ECS2 was launched during a time of increased demand in the bioenergy sector, leading to a greater interest in the scheme during its second phase before its end in 2015 (NNFCC, 2012). Although the schemes supported farmers with establishing costs, ongoing costs like harvesting and transport were not covered in either case (Glithero et al., 2013; Thornley, 2006). Previous surveys outlined similar barrier perceptions, as it forces a reliance on a limited number of purchasers and the small number of alternative markets for *Miscanthus* (Piterou et al., 2008; Sherrington et al., 2008), as well as being concerned about the security of demand to warrant the necessary long‐term commitment in related bioenergy sectors or companies (Piterou et al., 2008). The barriers determined in the present survey, supported by previous works, indicate that whilst there are no absolute barriers to bioenergy crop growing (McCormick & Kåberger, 2007), non‐technical challenges must be addressed to remove the perceived barriers farmers may have.

### 4.5 Benefits identified by respondents

The present work has outlined that 94% of respondents determined *Miscanthus* to having benefited their farming operations in some manner (Figure 7a). These benefits included the reliability of the UK market due to long‐term contracts with Terravesta and the improved profit margin (Figure 8a). The reliability of the market was previously noted as a strong reason for entering the *Miscanthus* growing market. Notably, from 2015 onward, Terravesta has registered increasing growers interest due to the stability of the market and also the improved planting of the rhizomes making for a more resilient crop, allowing for the establishment of long‐term contracts (Shepherd et al., 2020). In practical aspects, *Miscanthus* has reduced the need for spraying near playground and homes (Figure 8b). With the reduced spraying of chemical or biological fertilisers near communities, the risk of dermatological, gastrointestinal, neurological, carcinogenic, respiratory, reproductive and endocrine effects are significantly reduced (see e.g., Alewu & Nosiri, 2011; Mnif et al., 2011; WHO & UNEP, 1990). The reduced fertiliser reliance also reduces the run‐off into surface waters, thus reducing downstream effects on other ecosystems and could make *Miscanthus* an attractive crop to grow in nitrogen sensitive catchments which are numerous among clay soil valleys like those in the southwest of England.

Another benefit determined here was the crop allowing for the use of, marginal, remote, otherwise unusable, or applicable fields (Figure 8b). *Miscanthus* requires little input after the crop is established (two growing seasons), which minimises the labour requirements, water use, and the need for fertilisers and pesticides (Don et al., 2012; Heaton et al., 2004). Paradoxically this means that due to not requiring large spray booms, small or odd shaped fields or those with obstructions can be used. Previous works have further determined that the winter coverage of this perennial grass has positive effects on the soil quality (Hansen et al., 2004; Heaton et al., 2004). This was also noted as a benefit by survey participants, stating that reduced soil erosion was observed (Table 1). The crop was also found to benefit wildlife biodiversity (Bellamy et al., 2009; Bocquého & Jacquet, 2010; Semere & Slater, 2007a, 2007b). These were aspects that were ranked of high importance in making business decisions in the present survey (Figure 6b). This supports the alignment of this crop with the needs and ideals of the farmers.

### 4.6 Bioenergy policy landscape

Of the 8.5 million ha of land available for the growing of bioenergy crops (Lovett et al., 2014), only 96,000 ha were used for such crops in 2019. Of these 96,000 ha, *Miscanthus* accounted for 8000 ha, whereas maize and SRC woodlands accounted for 67,000 and 2000 ha, respectively (Bioenergy Insight, 2020). This means, that currently only approximately 1% of the available land is being used for bioenergy crops, of which *Miscanthus* made up less than 0.1% in 2019. However, to benefit from the benefits that perennial bioenergy crops such as *Miscanthus* have over annual crops, a stronger focus needs to be placed on a supporting policy framework.

The United Kingdom currently imports more than 60% of its heat and power biomass, as the demand of the current biomass power stations cannot be met by crops or trees grown in the United Kingdom. To determine the research needs, policy requirements and stakeholder uncertainties, a joint workshop was held in 2018. One issue that was highlighted was that the policy and regulatory framework in the United Kingdom, while successful at stimulating interest in bioenergy production, had now come to an end and future development is thus uncertain (Brown, 2019a). The future policy‐scene is thus unclear, posing a challenge to companies that are aiming to establish in the market and increase bioenergy crop production. It was determined that the expansion of the bioenergy sector is currently hampered by barriers of a lack of financial support for renewable heat generation compared with traditional fossil fuel use. Additionally, suggestions to restrict biomass heat plants to urban areas further limits the potential contribution of rurally produced biomass to the heat networks (Brown, 2019b). UK bioenergy is supplied mostly by liquid biofuels and plant biomass, which can include, for example, *Miscanthus*, but mostly uses wood and wood waste, general waste, animal biomass, as well as sewage and landfill gas (Brown, 2019a). This limits the potential for the bioenergy sector in the United Kingdom to increase by a factor of 2.5 by 2032 (Brown, 2019b), and to help meet various policy targets in the short and medium term, with technologies that are already available, in many cases offering low‐cost and renewable solutions (Brown, 2019a). Drawing a link to the current energy market and its responsiveness to political situations in other countries (e.g., Russia), the need for more self‐reliant markets is clear.

Suggestions for actions that would aid such developments include the introduction of a replacement for the current Renewable Heat Incentive (RHI), which was aimed at increasing the implementation of renewable heat technologies in households and businesses, including biomass pellet stoves and boilers for *Miscanthus* straw by providing financial incentives (DECC, 2015). The RHI further aimed to create a secure market for renewable heat technologies, supporting the development of Bioenergy with Carbon Capture Use and Storage (BECCUS), among others (Brown, 2019c), to which *Miscanthus* would further contribute to. Once an even policy field has been created, which supports the long‐term investment into bioenergy crops like *Miscanthus*, the perceived barriers to entry outlined previously can be addressed. With the proper recognition of the crop as a contributor to reaching the UK CCC's goals, and with the clear long‐term benefits of entering a stable market for bioenergy crops, more growers may be persuaded to grow *Miscanthus*, who might currently be convinced by the crop's biological benefits, but not the economic situation.

## 5 CONCLUSION

Although the survey is based on a limited number of participants, given the limited number of commercial growers currently in the United Kingdom, there are some indicators of farmer attitudes to *Miscanthus* from a number of these early adopters. For example, the survey has highlighted that the market for bioenergy crops must generate sufficient revenue, which must then be distributed accordingly among all actors in the value chain, to be profitable and lucrative. Although the crop itself was found to have only one perceived drawback, the difficulty in removing the crop (see question 30 and 31 in Material S2), the barriers to entry were almost exclusively centred around a lack of information and incentives. The recognition of the crops benefits and its role in reaching the UK's Committee on Climate change (UK CCC) targets would greatly drive forward the movement to recruiting new growers, as well as paving the way for the expansion of processing facilities. Future scientific research should thus strive to determine further benefits and uses of the crop and ensure adequate understanding of the environmental needs for successful growth. As the survey highlighted that the cooperation between the growers and the industry were good, more focus should be placed on direct dialogue with policy‐makers, taking the needs of growers and the concerns of stakeholders into consideration, to establish a bioenergy‐friendly future in the United Kingdom.

## CONFLICT OF INTEREST

The authors declare that they have no conflict of interest.

## Supporting information


Appendix S1

## Data Availability

The data have been uploaded to Dryad and can be accessed under: https://doi.org/10.5061/dryad.83bk3j9tz.

## References

[gcbb12997-bib-0001] Abram, M. (2021). How a carbon payment scheme will work for 100 UK farmers. Farmers Weekly. https://www.fwi.co.uk/news/environment/carbon/how‐a‐carbon‐payments‐scheme‐will‐work‐for‐100‐uk‐farmers

[gcbb12997-bib-0002] Adams, P. W. , Hammond, G. P. , McManus, M. C. , & Mezzullo, W. G. (2011). Barriers to and drivers for UK bioenergy development. Renewable and Sustainable Energy Reviews, 15(2), 1217–1227. 10.1016/j.rser.2010.09.039

[gcbb12997-bib-0003] Alewu, B. , & Nosiri, C. (2011). Pesticides and human health. In M. Stoytcheva (Ed.), Pesticides in the modern world—Effects of pesticides exposure. INTECH. 10.5772/18734

[gcbb12997-bib-0004] Anderson, E. , Arundale, R. , Maughan, M. , Oladeinde, A. , Wycislo, A. , & Voigt, T. (2011). Growth and agronomy of *miscanthus × giganteus* for biomass production. Biofuels, 2(2), 167–183. 10.4155/bfs.10.80

[gcbb12997-bib-0005] BEIS . (2021a). Net zero strategy: Build back greener. *Gov.Uk* (Issue October). https://www.gov.uk/government/publications/net‐zero‐strategy

[gcbb12997-bib-0006] BEIS . (2021b). The carbon capture and sorage infrastructure fund: An update on the design of the CCS infrastructuer fund . https://assets.publishing.service.gov.uk/government/uploads/system/uploads/attachment_data/file/984001/ccs‐infrastructure‐fund‐cif‐design.pdf

[gcbb12997-bib-0007] BEIS . (2022). Renewable electricity capacity and generation. https://www.gov.uk/government/statistics/energy‐trends‐section‐6‐renewables

[gcbb12997-bib-0008] Bellamy, P. E. , Croxton, P. J. , Heard, M. S. , Hinsley, S. A. , Hulmes, L. , Hulmes, S. , Nuttall, P. , Pywell, R. F. , & Rothery, P. (2009). The impact of growing *Miscanthus* for biomass on farmland bird populations. Biomass and Bioenergy, 33(2), 191–199. 10.1016/j.biombioe.2008.07.001

[gcbb12997-bib-0009] Bioenergy Insight . (2020). 96,000 hectares of UK agricultural land used for bioenergy crops in 2019. Bioenergy News. https://www.bioenergy‐news.com/news/96000‐hectares‐of‐uk‐agricultural‐land‐used‐for‐bioenergy‐crops‐in‐2019/

[gcbb12997-bib-0010] Bocquého, G. , & Jacquet, F. (2010). The adoption of switchgrass and *Miscanthus* by farmers: Impact of liquidity constraints and risk preferences. Energy Policy, 38(5), 2598–2607. 10.1016/j.enpol.2010.01.005

[gcbb12997-bib-0011] Brown, A. (2019a). Phase 1: Bioenergy in the UK—The state of play . https://www.r‐e‐a.net/wp‐content/uploads/2019/10/REA‐Bioenergy‐Strategy‐Phase‐1‐State‐of‐Play‐Web.pdf

[gcbb12997-bib-0012] Brown, A. (2019b). Phase 2: Bioenergy in the UK—A vision to 2032 and beyond . https://www.r‐e‐a.net/wp‐content/uploads/2019/10/REA‐Bioenergy‐Strategy‐Phase‐2‐A‐Vision‐to‐2032‐and‐Beyond.pdf

[gcbb12997-bib-0013] Brown, A. (2019c). Phase 3: Delivering the UK's bioenergy potential . https://www.bioenergy‐strategy.com/_files/ugd/be3f73_500621268fae43abb80388418f85a6ac.pdf

[gcbb12997-bib-0014] Carroll, A. , & Somerville, C. (2009). Cellulosic biofuels. Annual Review of Plant Biology, 60, 165–182. 10.1146/annurev.arplant.043008.092125 19014348

[gcbb12997-bib-0015] CCC . (2019). Annual report and accounts 2018–19. 10.4324/9781315148441-10

[gcbb12997-bib-0016] Clifton‐Brown, J. , Hastings, A. , Mos, M. , McCalmont, J. P. , Ashman, C. , Awty‐Carroll, D. , Cerazy, J. , Chiang, Y. C. , Cosentino, S. , Cracroft‐Eley, W. , Scurlock, J. , Donnison, I. S. , Glover, C. , Gołąb, I. , Greef, J. M. , Gwyn, J. , Harding, G. , Hayes, C. , Helios, W. , … Flavell, R. (2017). Progress in upscaling *Miscanthus* biomass production for the European bio‐economy with seed‐based hybrids. GCB Bioenergy, 9(1), 6–17. 10.1111/gcbb.12357

[gcbb12997-bib-0017] Clifton‐Brown, J. , Schwarz, K. U. , & Hastings, A. (2015). History of the development of *Miscanthus* as a bioenergy crop: From small beginnings to potential realisation. Biology and Environment, 115B(1), 1–13. 10.3318/BIOE.2015.05

[gcbb12997-bib-0018] Cross, S. , Welfle, A. J. , Thornley, P. , Syri, S. , & Mikaelsson, M. (2021). Bioenergy development in the UK & Nordic countries: A comparison of effectiveness of support policies for sustainable development of the bioenergy sector. Biomass and Bioenergy, 144(October 2020), 105887. 10.1016/j.biombioe.2020.105887

[gcbb12997-bib-0019] DECC . (2015). 2010 to 2015 government policy: Low carbon technologies. https://www.gov.uk/government/publications/2010‐to‐2015‐government‐policy‐low‐carbon‐technologies/2010‐to‐2015‐government‐policy‐low‐carbon‐technologies#appendix‐6‐renewable‐heat‐incentive‐rhi

[gcbb12997-bib-0020] DEFRA . (2003). Energy crop scheme—Establishment grant . http://adlib.everysite.co.uk/resources/000/030/119/grants_energy.pdf

[gcbb12997-bib-0021] DEFRA . (2019a). Crops grown for energy in the UK . 2017 (Issue January). https://assets.publishing.service.gov.uk/government/uploads/system/uploads/attachment_data/file/775243/nonfood‐statsnotice2017‐31jan19i.pdf

[gcbb12997-bib-0022] DEFRA . (2019b). The future farming and environment evidence compendium .

[gcbb12997-bib-0023] DEFRA . (2022). Farm business survey for England, Wales, and Northern Ireland . https://www.gov.uk/government/collections/farm‐business‐survey

[gcbb12997-bib-0024] Don, A. , Osborne, B. , Hastings, A. , Skiba, U. , Carter, M. S. , Drewer, J. , Flessa, H. , Freibauer, A. , Hyvönen, N. , Jones, M. B. , Lanigan, G. J. , Mander, Ü. , Monti, A. , Djomo, S. N. , Valentine, J. , Walter, K. , Zegada‐Lizarazu, W. , & Zenone, T. (2012). Land‐use change to bioenergy production in Europe: Implications for the greenhouse gas balance and soil carbon. GCB Bioenergy, 4, 372–391. 10.1111/j.1757-1707.2011.01116.x

[gcbb12997-bib-0025] Finch, J. W. , Karp, A. , McCabe, D. P. M. , Nixon, S. , Riche, A. B. , & Whitmore, A. P . (2009). Miscanthus, short‐rotation coppice and the historic environment .

[gcbb12997-bib-0026] Glithero, N. J. , Wilson, P. , & Ramsden, S. J. (2013). Prospects for arable farm uptake of short rotation coppice willow and *Miscanthus* in England. Applied Energy, 107(2013), 209–218. 10.1016/j.apenergy.2013.02.032 23825896 PMC3688319

[gcbb12997-bib-0027] Hansen, E. M. , Christensen, B. T. , Jensen, L. S. , & Kristensen, K. (2004). Carbon sequestration in soil beneath long‐term *Miscanthus* plantations as determined by 13C abundance. Biomass and Bioenergy, 26(2), 97–105. 10.1016/S0961-9534(03)00102-8

[gcbb12997-bib-0028] Harvey, J. (2007, January). A versatile solution? Growing *Miscanthus* for bioenergy. Renewable Energy World. https://www.renewableenergyworld.com/baseload/a‐versatile‐solution‐growing‐miscanthus‐for‐bioenergy‐51557/

[gcbb12997-bib-0029] Hastings, A. , Tallis, M. J. , Casella, E. , Matthews, R. W. , Henshall, P. A. , Milner, S. , Smith, P. , & Taylor, G. (2014). The technical potential of Great Britain to produce ligno‐cellulosic biomass for bioenergy in current and future climates. GCB Bioenergy, 6(2), 108–122. 10.1111/gcbb.12103

[gcbb12997-bib-0030] Heaton, E. A. , Clifton‐Brown, J. , Voigt, T. B. , Jones, M. B. , & Long, S. P. (2004). *Miscanthus* for renewable energy generation: European Union experience and projections for Illinois. Mitigation and Adaptation Strategies for Global Change, 9(4), 433–451. 10.1023/B:MITI.0000038848.94134.be

[gcbb12997-bib-0031] HM Government . (2020). The ten point plan for a green industrial revolution . https://assets.publishing.service.gov.uk/government/uploads/system/uploads/attachment_data/file/936567/10_POINT_PLAN_BOOKLET.pdf

[gcbb12997-bib-0032] House of Commons . (2018). UK progress on reducing nitrate pollution . https://publications.parliament.uk/pa/cm201719/cmselect/cmenvaud/656/656.pdf

[gcbb12997-bib-0033] Karp, A. , & Shield, I. (2008). Bioenergy from plants and the sustainable yield challenge. New Phytologist, 179(1), 15–32. 10.1111/j.1469-8137.2008.02432.x 18422906

[gcbb12997-bib-0034] Lewandowski, I. , Clifton‐Brown, J. C. , Andersson, B. , Basch, G. , Christian, D. G. , Jørgensen, U. , Jones, M. B. , Riche, A. B. , Schwarz, K. U. , Tayebi, K. , & Teixeira, F. (2003). Environment and harvest time affects the combustion qualities of *Miscanthus* genotypes. Agronomy Journal, 95(5), 1274–1280. 10.2134/agronj2003.1274

[gcbb12997-bib-0035] Lovett, A. , Sünnenberg, G. , & Dockerty, T. (2014). The availability of land for perennial energy crops in Great Britain. GCB Bioenergy, 6(2), 99–107. 10.1111/gcbb.12147

[gcbb12997-bib-0036] McCalmont, J. P. , Hastings, A. , McNamara, N. P. , Richter, G. M. , Robson, P. , Donnison, I. S. , & Clifton‐Brown, J. (2015). Environmental costs and benefits of growing *Miscanthus* for bioenergy in the UK. GCB Bioenergy, 9(3), 489–507. 10.1111/gcbb.12294 28331551 PMC5340280

[gcbb12997-bib-0037] McCormick, K. , & Kåberger, T. (2007). Key barriers for bioenergy in Europe: Economic conditions, know‐how and institutional capacity, and supply chain co‐ordination. Biomass and Bioenergy, 31(7), 443–452. 10.1016/j.biombioe.2007.01.008

[gcbb12997-bib-0038] Milner, S. , Holland, R. A. , Lovett, A. , Sunnenberg, G. , Hastings, A. , Smith, P. , Wang, S. , & Taylor, G. (2016). Potential impacts on ecosystem services of land use transitions to second‐generation bioenergy crops in GB. GCB Bioenergy, 8(2), 317–333. 10.1111/gcbb.12263 27547244 PMC4974899

[gcbb12997-bib-0039] Mnif, W. , Hassine, A. I. H. , Bouaziz, A. , Bartegi, A. , Thomas, O. , & Roig, B. (2011). Effect of endocrine disruptor pesticides: A review. International Journal of Environmental Research and Public Health, 8(6), 2265–2303. 10.3390/ijerph8062265 21776230 PMC3138025

[gcbb12997-bib-0040] NNFCC . (2012). Domestic energy crops: Potential and constraints review. https://assets.publishing.service.gov.uk/government/uploads/system/uploads/attachment_data/file/48342/5138‐domestic‐energy‐crops‐potential‐and‐constraints‐r.PDF

[gcbb12997-bib-0041] Piterou, A. , Shackley, S. , & Upham, P. (2008). Project ARBRE: Lessons for bio‐energy developers and policy‐makers. Energy Policy, 36(6), 2044–2050. 10.1016/j.enpol.2008.02.022

[gcbb12997-bib-0042] Semere, T. , & Slater, F. M. (2007a). Ground flora, small mammal and bird species diversity in *miscanthus* (*miscanthus×giganteus*) and reed canary‐grass (*Phalaris arundinacea*) fields. Biomass and Bioenergy, 31(1), 20–29. 10.1016/j.biombioe.2006.07.001

[gcbb12997-bib-0043] Semere, T. , & Slater, F. M. (2007b). Invertebrate populations in *Miscanthus* (Miscanthus×giganteus) and reed canary‐grass (*Phalaris arundinacea*) fields. Biomass and Bioenergy, 31(1), 30–39. 10.1016/j.biombioe.2006.07.002

[gcbb12997-bib-0044] Shepherd, A. , Clifton‐Brown, J. , Kam, J. , Buckby, S. , & Hastings, A. (2020). Commercial experience with *Miscanthus* crops: Establishment, yields and environmental observations. GCB Bioenergy, 12(7), 510–523. 10.1111/gcbb.12690

[gcbb12997-bib-0045] Sherrington, C. , Bartley, J. , & Moran, D. (2008). Farm‐level constraints on the domestic supply of perennial energy crops in the UK. Energy Policy, 36(7), 2504–2512. 10.1016/j.enpol.2008.03.004

[gcbb12997-bib-0047] Solarsense . (2022). Agriculture: Solar PV for farms, landowners and the agriculture sector. https://www.solarsense‐uk.com/filter_sector/agricultural/

[gcbb12997-bib-0048] Terravesta . (2021). Miscanthus research gets government funding to help the UK to meet net zero. Terravesta Times. https://www.terravesta.com/news/miscanthus‐research‐gets‐government‐funding‐to‐help‐the‐uk‐to‐meet‐net‐zero/

[gcbb12997-bib-0049] Terravesta . (2022). Current and future markets . https://www.terravesta.com/markets/#current‐and‐future‐markets

[gcbb12997-bib-0050] Thornley, P. (2006). Increasing biomass based power generation in the UK. Energy Policy, 34(15), 2087–2099. 10.1016/j.enpol.2005.02.006

[gcbb12997-bib-0051] UK Government . (2008). UK Climate Change Act . https://www.legislation.gov.uk/ukpga/2008/27/contents/enacted

[gcbb12997-bib-0052] UK Government . (2019). UK Climate Change Act 2008 (2050 Target Amendment) Order 2019 .

[gcbb12997-bib-0053] WECA . (2020). West of England Climate Emergency Action Plan . https://westofengland‐ca.moderngov.co.uk/documents/s2200/CEActionPlan.pdf

[gcbb12997-bib-0054] WHO, & UNEP . (1990). Public health impact of pesticides used in agriculture . https://apps.who.int/iris/handle/10665/39772

[gcbb12997-bib-0055] World Bank Group . (2021). Power purchase agreements (PPAs) and energy purchase agreements (EPAs) . https://ppp.worldbank.org/public‐private‐partnership/sector/energy/energy‐power‐agreements/power‐purchase‐agreements

