# Supplementary material for: Expanding the Miscanthus market in the UK: Growers in profile and experience, benefits and drawbacks of the bioenergy crop
Source: Glob Change Biol Bioenergy. 2022 Sep 14;14(11):1205–18. doi: 10.1111/gcbb.12997 (PMC9825852; doi:10.1111/gcbb.12997)
Supplement: Supplementary file 1 — Appendix S1 [file GCBB-14-1205-s001.zip › GCBB_12997_Supplementary_Material_Miscanthus_GCB.docx]

# **Supplementary Material**

## **Supplementary Material 1**

***Miscanthus* survey**

**Grower experience with *Miscanthus***

Carried out by the University of Aberdeen in conjunction with Terravesta.

The survey should take 15 minutes to complete. Could you please return your questionnaire before the end of October.
At the end of the survey, once the submit button is pressed, you will be taken out of the survey to a completely separate document where you can optionally provide your email address for a draw for a Wensleydale cheese hamper.

We would like to know why a grower plants *Miscanthus* and what, if any, benefits or drawbacks it brings to their farm business and to identify the barriers to expanding the *Miscanthus* cropping area in the UK. Results of this questionnaire will be completely anonymous, with no contact details, no growers will be identified. Anonymous questionnaires will be stored on secure computer storage at the University of Aberdeen which will be destroyed after three years. Your participation is purely voluntary, and you may withdraw from the research at any time. Collective statistics from all the anonymous survey responses will be used in reports and summaries, which will be available to other researchers and the industry and to inform policy advice.
Email [Anita.Shepherd@abdn.ac.uk](mailto:Anita.Shepherd@abdn.ac.uk) for questions on completing this survey.

Please tick the box below to show you consent for your data be used in this way. If the consent box is not ticked, the questionnaire will be deleted and of no use.
Questions are divided into sections
- About the farm
- About the grower
- Benefits and drawbacks of *Miscanthus*

Question 1: Do you consent to the data from this survey being used anonymously in reports and summaries?

Yes
No

**About the farm**

Question 2: What type of farm(s) do you have? Please tick all that apply.

- Arable
- Horticulture
- Grass/pasture
- Woody plants
- Livestock
- Energy related
- Rather not answer
- Other

Of other, please specify and add its area in hectares

Question 3: How many hectares of Arable? (Write number only)

Question 4: How many hectares of Horticulture? (Write number only)

Question 5: How many hectares of Grass/pasture? (Write number only)

Question 6: How many hectares of Woody plants? (Write number only)

Question 7: How many hectares of Livestock? (Write number only)

Question 8: Which type of land use/crop/activity related to energy do you have? (For example: *Miscanthus*, short rotation coppiced willow, biodigestor, solar, wind, etc.)

Question 9: How many hectares of agriculture related to energy

Question 10: Based on the Farm Business Survey for England, Wales, and N. Ireland definition, what size would you classify your whole farm business?

- Small (labour required is less than 3 full-time workers)
- Medium (labour required is 2 but less than 3 full-time workers)
- Large (labour required is 3 but less than 5 full-time workers)
- V. large (labour required is 5 or more full-time workers)
- Rather not say

Question 11: How many hectares is your total *Miscanthus* cropping area (all fields added together)?

- Less than 5 ha (less than 12 acres)
- 5 ha to 9 ha (12 to 22 acres)
- 10 ha to 14 ha (24 to 34 acres)
- 15 ha to 19 ha (37 to 47 acres)
- 20 ha or over (49 acres or over)
- Don’t know

Question 12: Which year approximately did you first plant *Miscanthus*?

- Prior to 2004
- 205-2009
- 2010-2014
- 2015-2019
- Don’t know

Question 13: Before you planted the *Miscanthus* what was the last use of the field(s)?

- Fallow
- Hay/silage
- Pasture/livestock
- Other fodder crop
- Arable rotations
- Horticulture
- Other vegetables/fruit
- Hops
- Wood/shrubs
- Other

If other, please specify

Question 14: Which crops did you grow in rotation?

Question 15: If you had not planted *Miscanthus*, what would you have planted or used the field(s) for?

Question 16: Do you

- Implement some agro-ecological strategies (e.g., edge row or field margins with beekeeping)?
- Farm organically?
- Take part in any environmental schemes?
- Was your farm in the Less Favourable Area Support Scheme under CAP?
- None of these
- Rather not say

Question 17: Do you have any other renewables (solar, wind, biodigestor crops, etc.)?

If so, which type?

**About the grower**

Question 18: What age is the business decision-maker?

- Under 25
- 26-40
- 41-59
- 60+
- Rather not say

Question 19: Do you have a post-school educational qualification (diploma, certificate, degree, etc.)?

- Yes
- No
- Rather not say

Question 20: On a scale of 1-5 for each item, 5 being very important to 1 being not important, or not applicable (N/A), how much do you value these/how important are these farming decisions you take?

|  | 1 | 2 | 3 | 4 | 5 | N/A | Rather not say |
| --- | --- | --- | --- | --- | --- | --- | --- |
| A transition to green energy |  |  |  |  |  |  |  |
| Combatting climate change |  |  |  |  |  |  |  |
| Biodiversity |  |  |  |  |  |  |  |
| Healthy pollinators |  |  |  |  |  |  |  |
| More wildlife in your fields |  |  |  |  |  |  |  |
| Soil health |  |  |  |  |  |  |  |
| Reduced concentrations of agrichemicals in soil and water |  |  |  |  |  |  |  |

**Benefits and drawbacks of *Miscanthus***

Question 21: Have you found *Miscanthus* to be beneficial to your business?

- Yes
- No
- Don’t know
- Rather not say

Question 22: What problems have your found with *Miscanthus*, please answer on a scale of 1-5 for each item, 5 being very important to 1 being not important

|  | 1 | 2 | 3 | 4 | 5 | N/A | Rather not say |
| --- | --- | --- | --- | --- | --- | --- | --- |
| Yields were lower than expected/underperformed |  |  |  |  |  |  |  |
| Loss of flexibility in reacting to markets by changing crop type |  |  |  |  |  |  |  |
| Immature market for M*iscanthus* straw |  |  |  |  |  |  |  |
| Increase of pest species |  |  |  |  |  |  |  |
| Not found problems |  |  |  |  |  |  |  |

Question 23: If there are any other reasons you answered ’no’ to *Miscanthus* being beneficial other than above, please tell us

Question 24: On a scale of 1-5 for each item, 5 being very important to 1 being not important, how has *Miscanthus* helped your farm economy

|  | 1 | 2 | 3 | 4 | 5 | N/A | Rather not say |
| --- | --- | --- | --- | --- | --- | --- | --- |
| Improved profit margins in comparison to growing alternative crops on that field |  |  |  |  |  |  |  |
| Increased profit margin of the farm as a whole |  |  |  |  |  |  |  |
| Reliable market |  |  |  |  |  |  |  |
| *Miscanthus* has not helped my farm economy |  |  |  |  |  |  |  |

Question 25: If there are any other reasons *Miscanthus* has helped your farm economy, please tell us

Question 26: On a scale of 1-5 for each item, 5 being very important to 1 being not important, how has *Miscanthus* helped the practical aspect of farming

|  | 1 | 2 | 3 | 4 | 5 | N/A | Rather not say |
| --- | --- | --- | --- | --- | --- | --- | --- |
| Enabled cropping in remote fields/difficult to access fields |  |  |  |  |  |  |  |
| Reduced spraying near houses/playgrounds |  |  |  |  |  |  |  |
| Reduced crop vandalism |  |  |  |  |  |  |  |
| *Miscanthus* has not helped the practical aspects of farming |  |  |  |  |  |  |  |

Question 27: If there are any other ways *Miscanthus* has helped the practical aspect of farming, please tell us

Question 28: Were there any particular benefits of having *Miscanthus* during the time of:

|  | Yes | No | No difference/not noticed |
| --- | --- | --- | --- |
| Brexit |  |  |  |
| Covid-19 restrictions |  |  |  |

Question 29: If you answered yes to Brexit or Covid-19, how did *Miscanthus* help

- Secured contract with end-user (reliable market)
- Reduced need for hired labour
- More time to spend on other matters
- Did not answer yes to benefits of *Miscanthus* during Brexit or Covid-19

Other, please specify

Question 30: Thinking back to when you first invested in *Miscanthus*, do you think you had enough advice on growing the crop?

- Yes
- No
- No opinion
- Rather not say

Question 31: If you ever removed a *Miscanthus* crop, how did you find the operation?

- Difficult
- Not difficult
- Never removed a *Miscanthus* crop
- No opinion
- Rather not say

Question 32: If you were left with any field or crop problems after *Miscanthus* removal, please specify?

Question 33: On a scale of 1-5 for each item, 5 being very important to 1 being not important, what did you think is the largest barrier to expanding *Miscanthus* cropping area?

|  | 1 | 2 | 3 | 4 | 5 | N/A | Rather not say |
| --- | --- | --- | --- | --- | --- | --- | --- |
| Cost of establishing |  |  |  |  |  |  |  |
| Ongoing management costs |  |  |  |  |  |  |  |
| Availability of suitable land |  |  |  |  |  |  |  |
| Limited understanding of the crop within the wider farmer community |  |  |  |  |  |  |  |
| Crop vandalism |  |  |  |  |  |  |  |
| Trust of crop/company |  |  |  |  |  |  |  |
| Environmental: Increase of pest species/water scarcity |  |  |  |  |  |  |  |
| Use of contractors |  |  |  |  |  |  |  |

Question 34: If you see a different barrier than the above to expanding *Miscanthus*, please tell us

Question 35: If the same amount was given as a subsidy for growing *Miscanthus*, would you rather it was paid

- As a planting grant
- Annually at harvest
- No preference
- Rather not say

Question 36: What is the main one or two characteristics that you think could increase *Miscanthus* production across the UK? (Open question, can include crop breeding characteristics, distribution, agronomy, government incentives, etc.)

Question 37: Would you ever consider expanding your cropping area of *Miscanthus* in the future?

- Yes
- No
- Don’t know
- Rather not say

Results from this questionnaire will help us discover growers’ experience on the benefits of and barriers to growing *Miscanthus* and why they chose to grow it. All data you have provided will be collected with other growers’ surveys and converted into statistics and will be anonymous.

Please press the submit button, and you will be taken to a separate document where you can optionally add your email address to be entered into a draw for a Wensleydale cheese hamper. Could you please return your questionnaire before the end of October.

Thank you for your time in completing this survey.

Please click submit to send your replies.

**Supplementary Material 2**

***Miscanthus* survey responses**

Only surveys with “Yes” as answer to question 1 are presented below (n=17).

**About the farm**

| **ID** | **Q2-7, 9** | **Q8** | **Q10** | **Q11** | **Q12** | **Q13** | **Q14** |
| --- | --- | --- | --- | --- | --- | --- | --- |
| **1** | Arable (220), grass/pasture (80), woody plants (40), energy related (2) | *Miscanthus*, willow | Medium | >20ha | 2005-2009 | Pasture/livestock, arable rotations | Cereals |
| **2** | Grass/pasture (20), livestock (20), energy related (42) | *Miscanthus* | Small | >20ha | 2015-2019 | Pasture/livestock |  |
| **3** | Mixed without livestock | *Miscanthus* | Small | 10-14ha | 2005-2009 | Arable rotations | Wheat, maize |
| **4** | Mixed | *Miscanthus* | Small | >20ha | Don’t know | Arable rotations | Wheat, barley, OSR |
| **5** | Arable (300) | *Miscanthus* | Medium | 15-19ha | 2005-2009 | Arable rotations | Wheat, barley, OSR, sugar beet |
| **6** | Arable (800) | *Miscanthus*, solar | Medium | 5-9ha | <2004 | Arable rotations | Wheat, barley, potatoes |
| **7** | Arable (90), grass/pasture (6), livestock (6), energy related (37) | *Miscanthus*, solar | Small | >20ha | <2004 | Arable rotations | Barley, rye |
| **8** | Arable (ND) |  | Small | 5-9ha | 2010-2014 | Arable rotations | Wheat, OSR, beans |
| **9** | Arable (65), grass/pasture (15), energy related (16) | *Miscanthus*, willow, solar, SRC, biomass heating | Small | 5-9ha | 2005-2009 | Arable rotations | Wheat, barley, OSR |
| **10** | Arable (200), energy related (14) | *Miscanthus* | Medium | 10-14ha | 2010-2014 | Arable rotations | Wheat, barley, OSR |
| **11** | Arable (450) | Miscanthus, solar, wind | Medium | 5-9ha | 2015-2019 | Arable rotations | Wheat, OSR |
| **12** | Arable (80), grass/pasture (20), energy related (23) | *Miscanthus* (4ha), wind (19ha) | Medium | <5ha | <2004 | Fallow |  |
| **13** | Energy related (45) | *Miscanthus* | Small | >20ha | 2015-2019 | Hay/silage, arable rotations | Feed wheat, grass for silage for dairy enterprise |
| **14** | Grass/pasture (13) | *Miscanthus* | Small | 5-9ha | 2005-2009 | Arable rotations | Wheat grass |
| **15** | Horticulture (20) | *Miscanthus* | Very large | 10-14ha | 2015-2019 | Fallow, other fodder crop |  |
| **16** | Arable (250) | *Miscanthus* | Small | 10-14ha | 2015-2019 | Arable rotations | Continuous wheat |
| **17** | Arable (250) | *Miscanthus* | Small | 15-19ha | 2015-2019 | Arable rotations | Wheat, peas |

| **ID** | **Q15** | **Q16** | **Q17** |
| --- | --- | --- | --- |
|  |  |  |  |
| **1** | Cereal, grass | Environmental schemes | Yes (Biomass) |
| **2** | Grazing grass | Agro-ecological strategies, environmental schemes | No |
| **3** | Grazing cattle | Agro-ecological strategies | No |
| **4** | Wheat, barley, OSR | Agro-ecological strategies | No |
| **5** | Wheat, barley, OSR, sugar beet | Agro-ecological strategies, environmental schemes | Yes (Wind, solar) |
| **6** | Grass | Agro-ecological strategies, environmental schemes | Yes (Solar) |
| **7** | Cereals | Agro-ecological strategies | Yes (Solar, ground source heating) |
| **8** | Arable rotation | Less Favourable Area Support Scheme (CAP) | No |
| **9** | Wheat, barley, OSR | Environmental schemes | Yes (solar, biomass heating) |
| **10** | Wheat, OSR | Agro-ecological strategies, environmental schemes | No |
| **11** | Arable | Agro-ecological strategies, environmental schemes | Yes (Wind, solar) |
| **12** | Grass | Environmental schemes | Yes (Solar) |
| **13** |  | Environmental schemes | No |
| **14** | Wheat grass | Agro-ecological strategies | No |
| **15** | Hemp, pulses, willow, flax (experimental) | Agro-ecological strategies | No |
| **16** | Continuous wheat | Agro-ecological strategies | No |
| **17** | Wheat, peas | Agro-ecological strategies | No |

**About the grower**

| **ID** | **Q18** | **Q19** | **Q20** | | | | | | |
| --- | --- | --- | --- | --- | --- | --- | --- | --- | --- |
|  |  |  | **Green energy** | **Climate change** | **Biodiversity** | **Healthy pollinators** | **More wildlife** | **Soil health** | **Agri-chemicals** |
| **1** | 41-59 | Yes | 4 | 4 | 4 | 4 | 4 | 4 | 4 |
| **2** | 41-59 | Yes | 5 | 5 | 5 | 5 | 5 | 5 | 5 |
| **3** | >60 | Yes | 5 | 5 | 5 | 5 | 5 | 5 | 5 |
| **4** | >60 | Yes | 4 | 4 | 4 | 4 | 4 | 4 | 4 |
| **5** | >60 | Yes | 2 | 3 | 3 | 3 | 3 | 4 | 1 |
| **6** | Rather not say | Yes | 3 | 4 | 4 | 4 | 4 | 5 | 4 |
| **7** | >60 | Yes | 3 | 4 | 3 | 4 | 2 | 4 | 4 |
| **8** | >60 | Yes | 4 | 4 | 5 | 5 | 5 | 5 | 4 |
| **9** | 41-59 | Yes | 4 | 4 | 3 | 1 | 1 | 5 | 4 |
| **10** | >60 | Yes | 4 | 4 | 4 | 4 | 4 | 4 | 4 |
| **11** | 41-59 | Yes | 4 | 5 | 4 | 5 | 5 | 4 | 5 |
| **12** | 41-59 | Yes | 4 | 4 | 5 | 5 | 5 | 5 | 5 |
| **13** | >60 | Yes | 5 | 5 | 4 | 3 | 5 | 4 | 5 |
| **14** | >60 | Yes | 5 | 5 | 5 | 5 | 5 | 5 | 5 |
| **15** | 26-40 | No | 5 | 5 | 4 | 4 | 4 | 4 | 4 |
| **16** | 26-40 | No | 2 | 3 | 5 | 5 | 5 | 5 | 5 |
| **17** | 41-59 | Yes | 5 | 3 | 4 | 5 | 3 | 5 | 3 |

**Benefits and drawbacks of *Miscanthus***

| **ID** | **Q21** | **Q22** | | | | | **Q24** | | | | **Q25** |
| --- | --- | --- | --- | --- | --- | --- | --- | --- | --- | --- | --- |
|  |  | **Lower yield** | **Loss of flexibility** | **Immature market** | **Pest species** | **None** | **Profit margin (fields)** | **Profit margin (farm)** | **Reliable market** | **None** |  |
| **1** | Yes |  |  |  |  |  | 2 | 2 | 2 | 2 |  |
| **2** | Yes |  |  |  |  |  | 4 | 3 | 4 | 1 | Freed up workload |
| **3** | Yes |  |  |  |  |  | 5 | 5 | 5 | N/A | Low labour needed |
| **4** | Yes |  |  |  |  |  | 4 | 4 | 4 | 1 |  |
| **5** | Yes |  |  |  |  |  | 4 | 2 | 3 | 1 |  |
| **6** | Yes |  |  |  |  |  | 4 | 2 | 2 | 3 | Use of small fields |
| **7** | Yes |  |  |  |  |  | 5 | 4 | 4 |  |  |
| **8** | Yes |  |  |  |  |  |  |  |  | 5 |  |
| **9** | Yes |  |  |  |  |  | 2 | 2 | 2 | N/A |  |
| **10** | Yes |  |  |  |  |  | 5 | 4 | 5 | N/A | Spread risk guaranteed market |
| **11** | Yes |  |  |  |  |  | 3 | 3 | 5 | 2 |  |
| **12** | Yes |  |  |  |  |  | 3 | 3 | 2 | 3 |  |
| **13** | Yes |  |  |  |  |  | 4 | 4 | 4 | N/A | Low maintenance crop, good on wet heavy land |
| **14** | Yes |  |  |  |  |  | 5 |  |  |  |  |
| **15** | No | 5 | 4 | 4 | 4 | 4 |  |  |  |  |  |
| **16** | Don’t know |  | 3 | 3 | 2 | 1 |  | 3 | 5 | N/A |  |
| **17** | Don’t know |  | 3 | 3 | 3 | 5 |  |  |  | 3 |  |

| **ID** | **Q26** | | | | **Q27** | **Q28** | | **Q29** | **Q30** | **Q31, 32** |
| --- | --- | --- | --- | --- | --- | --- | --- | --- | --- | --- |
|  | **Use of remote field** | **Reduced spraying** | **Reduced vandalism** | **None** |  | **Brexit** | **Covid-19** |  |  |  |
| **1** | 2 | 2 | 2 | 4 | Less inputs | No | No | Did not harvest due to covid-19 | Yes | Never removed |
| **2** | 3 | 3 | 2 | 1 |  | Yes | Yes | Reliable market, more time for other tasks | No | Difficult |
| **3** | 5 | 5 | 4 | N/A | Contractor/ renting for inexperienced owners | Yes | Yes | Reliable market | Yes | Never removed |
| **4** | 3 | 1 | 1 | N/A |  | No | No |  | No | Never removed |
| **5** | 4 | N/A | N/A | 2 |  | No | No |  | Yes | Never removed |
| **6** | 4 | 3 | 3 | 1 |  | No effect | No effect |  | No | Never removed |
| **7** | 1 | 2 | 1 | ND | Stopped annual erosion of blow-away sand. Greatly reduced chemical and fertiliser use | No effect | No effect |  | No | Difficult. It takes a long time to die |
| **8** | 4 | 5 | N/A | 3 |  | No | No |  | Yes |  |
| **9** | 1 | 1 | 1 | N/A |  | No effect | No effect |  | No | Never removed |
| **10** | 5 | N/A | N/A | N/A |  | No effect | No effect |  | Yes | Never removed |
| **11** | 5 | 2 | 2 | 2 |  | No | No |  | Yes | Never removed |
| **12** | 1 | N/A | 4 | 4 |  | No effect | No effect |  | Yes | Never removed |
| **13** | 3 | 4 | 1 | N/A | Reduced run-off of heavy rain and soil | No effect | No effect |  | Yes | Never removed |
| **14** | 5 | N/A | N/A | 5 |  | Yes | Yes | Reliable market | No | Never removed |
| **15** |  |  |  |  |  |  |  |  | Yes | Difficult |
| **16** |  | 5 | 5 | 3 |  | No effect | Yes | Reduced need for labour, more time for other tasks | Yes | Never removed |
| **17** |  | 5 | N/A | 5 |  | No effect | No effect |  | Yes | Never removed |

| **ID** | **Q33** | | | | | | | | **Q34** | **Q35** |
| --- | --- | --- | --- | --- | --- | --- | --- | --- | --- | --- |
|  | **Establishing cost** | **Management cost** | **Suitable land** | **Knowledge gaps** | **Crop vandalism** | **Crop/ company** | **Environmental** | **Use of contractor** |  |  |
| **1** | 3 | 3 | 3 | 3 | 3 | 3 | 3 | 3 |  | Annual |
| **2** | 3 | 3 | 5 | 3 | 2 | 2 | 2 | 2 |  | Annual |
| **3** | 5 | 1 | 1 | 1 | 1 | 1 | 1 | 1 |  | Grant |
| **4** | 4 | Unknown | N/A | N/A | N/A | 3 | N/A | N/A |  | No preference |
| **5** | 5 | 2 | 3 | 1 | 1 | 3 | 2 | 3 |  | Grant |
| **6** | 3 | 2 | 4 | 2 | 1 | 2 | 3 | 3 | Landlord/shoot | No preference |
| **7** | 2 | 2 | 3 | 4 | 1 | 4 | 1 | 2 | Changing contracts, delayed payment | Grant |
| **8** | 5 | 3 | 3 | 3 | N/A | 3 | 4 | 4 |  | Grant |
| **9** | 4 | 1 | 3 | 2 | 1 | 3 | 1 | 1 | Cost of road haulage | Rather not say |
| **10** | 4 | 3 | 3 | 4 | 2 | 1 | 4 | 3 |  | Annual |
| **11** | 5 | 2 | 2 | 3 | 1 | 4 | 4 | 3 |  | Annual |
| **12** | 5 | 3 | 3 | N/A | 4 | 4 | 4 | 4 |  | Grant |
| **13** | 5 | 1 | 3 | 3 | 1 |  | 2 | 4 | Limited market means high transport costs for certain regions. Research on alternative uses and distributed processing is very important. |  |
| **14** | 5 | 4 | 5 | 5 | 3 | 5 | 3 | 5 |  | Grant |
| **15** | 5 | 5 | 4 | 4 | 4 | 4 | 4 | 4 |  | Grant |
| **16** | 5 | 2 | 4 | 3 | 3 | Unknown | 3 | 4 |  | Annual |
| **17** | 1 | 3 | 3 | 3 | N/A | N/A | N/A | 3 |  | Annual |

| **ID** | **Q26** | **Q37** |
| --- | --- | --- |
| **1** | Carbon storage | No |
| **2** | More outlets | Yes |
| **3** | Ignorance of advantages; not mentioned by DEFRA/RPA – seems useless; farmers have no first/second hand experience and are very wary of change | Yes |
| **4** | Government incentives | Don’t know |
| **5** |  | Yes |
| **6** | Price (even after growing costs), especially in relation to cereals | Yes |
| **7** |  | No |
| **8** |  | Don’t know |
| **9** | More local markets | Yes |
| **10** | More knowledge about crop carbon capture | Yes |
| **11** | Government incentives | Yes |
| **12** | High prices for the bales; not such a complicated price formulation | Don’t know |
| **13** | Recognition as carbon negative; incentivise growers (monetise carbon capture and sequestration); research on the long-term soil health benefits, extend maximum yields over extended lifetimes; bigger selection of higher yielding varieties to fit different soil conditions | Yes |
| **14** | Appreciation of minimal herbicide, pesticide, and tractor use; green credentials | Yes |
| **15** |  | Yes |
| **16** | Government incentives | Yes |
| **17** |  | Yes |
